# Obesity-related DNA methylation at imprinted genes in human sperm: Results from the TIEGER study

**DOI:** 10.1186/s13148-016-0217-2

**Published:** 2016-05-06

**Authors:** Adelheid Soubry, Lisa Guo, Zhiqing Huang, Cathrine Hoyo, Stephanie Romanus, Thomas Price, Susan K. Murphy

**Affiliations:** Epidemiology Research Group, Department of Public Health and Primary Care, Faculty of Medicine, KU Leuven University, 3000 Leuven, Belgium; Department of Obstetrics and Gynecology, Division of Gynecologic Oncology, Duke University Medical Center, Durham, NC 27708 USA; Department of Biological Sciences, Center for Human Health and the Environment, North Carolina State University, Raleigh, NC 27633 USA; Department of Obstetrics and Gynecology, Division of Reproductive Endocrinology and Fertility, Duke University Medical Center, Durham, NC 27713 USA; Duke Cancer Institute, Duke University School of Medicine, Durham, NC 27710 USA

**Keywords:** Epigenetics, Sperm, Obesity, TIEGER study, Imprinted gene, Methylation

## Abstract

**Background:**

Epigenetic reprogramming in mammalian gametes resets methylation marks that regulate monoallelic expression of imprinted genes. In males, this involves erasure of the maternal methylation marks and establishment of paternal-specific methylation to appropriately guide normal development. The degree to which exogenous factors influence the fidelity of methylation reprogramming is unknown. We previously found an association between paternal obesity and altered DNA methylation in umbilical cord blood, suggesting that the father’s endocrine, nutritional, or lifestyle status could potentiate intergenerational heritable epigenetic abnormalities. In these analyses, we examine the relationship between male overweight/obesity and DNA methylation status of imprinted gene regulatory regions in the gametes.

**Methods:**

Linear regression models were used to compare sperm DNA methylation percentages, quantified by bisulfite pyrosequencing, at 12 differentially methylated regions (DMRs) from 23 overweight/obese and 44 normal weight men. Our study population included 69 volunteers from The Influence of the Environment on Gametic Epigenetic Reprogramming (TIEGER) study, based in NC, USA.

**Results:**

After adjusting for age and fertility patient status, semen from overweight or obese men had significantly lower methylation percentages at the *MEG3* (β = −1.99; SE = 0.84; *p* = 0.02), *NDN* (β = −1.10; SE = 0.47; *p* = 0.02), *SNRPN* (β = −0.65; SE = 0.27; *p* = 0.02), and *SGCE/PEG10* (β = −2.5; SE = 1.01; *p* = 0.01) DMRs. Our data further suggest a slight increase in DNA methylation at the *MEG3-IG* DMR (β = +1.22; SE = 0.59; *p* = 0.04) and *H19* DMR (β = +1.37; SE = 0.62; *p* = 0.03) in sperm of overweight/obese men.

**Conclusions:**

Our data support that male overweight/obesity status is traceable in the sperm epigenome. Further research is needed to understand the effect of such changes and the point of origin of DNA methylation differences between lean and overweight/obese men. Together with our earlier reports on paternal obesity and epigenetic shifts in the offspring, our studies set the groundwork for future studies investigating male gametic methylation aberrations due to paternal lifestyle factors such as obesity.

**Electronic supplementary material:**

The online version of this article (doi:10.1186/s13148-016-0217-2) contains supplementary material, which is available to authorized users.

## Background

The body of research supporting the hypothesis that adult chronic diseases, such as type II diabetes, metabolic disorders and obesity, have early-life origins has grown into the emergent field of developmental origins of health and disease (DOHaD) [1]. In light of the rising obesity epidemic, parental obesity’s role in influencing offspring development has become of particular interest [2, 3]. The relationship between maternal influences related to diet and obesity and offspring outcome has been noted in many studies [4–6], but there has been comparatively less research on the role of fathers. However, a growing consensus agrees that the influence of paternal factors, particularly obesity, on offspring health should not be overlooked [3, 7–9].

Animal models have shown that paternal obesity is associated with negative outcomes in offspring, including increased risk of obesity, metabolic syndrome, and subfertility [8, 10, 11]. While intergenerational relationships are more difficult to assess in humans, we recently reviewed a number of studies that linked paternal nutrition or obesity to offspring outcome [12]. Paternal obesity is an independent risk factor for autism spectrum disorder [13]. Paternal body fat composition and paternal grandfather nutritional status have also been linked to body fat in daughters and the mortality risk ratio of grandchildren, respectively [14, 15]. The observed transgenerational impact of paternal obesity may be due to epigenetic rather than genetic changes [7, 16, 17]. The epigenome encompasses a regulatory and memory function, mediated by DNA methylation, histone modifications, and non-coding RNAs. It is responsible for somatically heritable changes in gene function without a change in DNA sequence [18, 19]. The epigenome can be altered in response to environmental insults, such as obesity, diet, and toxins [3]. This plasticity potentially allows epigenetic changes due to environmental factors to occur in the (male) germ line and be passed to offspring [12]. Accordingly, significant offspring epigenetic changes have been linked to paternal obesity and nutritional status in numerous animal studies [10, 20–23].

Our prior work on the Newborn Epigenetic STudy (NEST) cohort showed that newborns of obese fathers have a modified epigenome in the form of altered methylation profiles at some imprinted gene regulatory regions [24, 25]. Imprinted genes differ from other epigenetically regulated genes in that their epigenetic marks and expression patterns are parent-of-origin-dependent, and these patterns are transferred with fidelity and consistency resulting in monoallelic expression [26, 27]. Imprint marks resist the global epigenetic reprogramming that occurs after fertilization, prior to germ layer specification, effectively allowing offspring to inherit any alterations to  these stable methylation marks from parents [7]. Thus, imprinted genes are ideal candidates to investigate early-acquired paternal contributions to offspring epigenomes.

In order to definitively link paternal obesity to offspring methylation modifications, such changes must be traced back to the semen of the father. Examination of sperm cells allows for the study of possible changes in the establishment of the male gamete epigenome prior to any influence and modulation from maternal factors and the *in utero* environment. Animal studies have already begun to link offspring epigenetic and phenotypic abnormalities to corresponding paternal sperm epigenetic changes. Wei et al. found that male mice with induced insulin resistance and impaired fasting glucose had offspring with increased susceptibility to diabetes and altered methylation patterns in their pancreatic islets that matched altered methylation in sperm from their fathers [20].

In human populations, very little research has been done linking paternal obesity to specific epigenetic modifications in sperm. The aim of this study was to determine whether or not there is a relationship between overweight/obesity status and methylation profiles in sperm at 12 imprinted regions. Our genes of interest include maternally expressed gene 3 (*MEG3*), paternally expressed gene 1/mesoderm specific transcript (*PEG1*/*MEST*), insulin-like growth factor 2 (*IGF2*), *H19*, growth factor receptor-bound protein 10 (*GRB10*), neuronatin (*NNAT*), necdin (*NDN*), small nuclear ribonucleoprotein polypeptide N (*SNRPN*), epsilon sarcoglycan (*SGCE*)/paternally expressed gene 10 (*PEG10*), paternally expressed gene 3 (*PEG3*), and pleiomorphic adenoma gene-like 1 (*PLAGL1*). These imprinted genes are growth effectors, important in early embryonic and fetal growth and/or regulation of tumor growth.

## Methods

### Study participants and data collection

Healthy male participants were recruited as part of The Influence of the Environment on Gametic Epigenetic Reprogramming Male (TIEGER) study, a larger project aimed at investigating associations between the sperm epigenome and various factors, including obesity and toxic environmental exposures. Participants were recruited through internet advertisements and flyers distributed within the city limits of Durham, NC from May 2012 to November 2013. Eligibility criteria included self-reported Caucasian ethnicity/race, non-smoking, no personal history of cancer, no vasectomy or other procedures that could cause infertility, and 18–35 years of age. A total of 81 men expressed interest in participating in the study. Six men were excluded because they were non-Caucasian. Six men declined participation prior to informed consent, resulting in 69 Caucasian men completing the study. Participants were required to come in for one visit at the Duke Fertility Center, Durham, NC. Because recruitment took place at this clinic, couples attending the clinic were also asked to participate, provided that men were not suspected of having underlying male fertility issues. Patient status (yes or no) was recorded. A clinical research nurse measured height and weight, and body mass index (BMI) was calculated as kilogram per square meter (kg/m^2^). The subjects were asked to complete a short questionnaire soliciting information on socio-demographics and lifestyle factors, including level of education, marital status, number of children fathered, occupation, and physical activity. Two men had insufficient sperm counts; hence, DNA methylation analyses were obtained for 67 of 69 Caucasian participants.

### Specimen collection

Volunteers were asked to donate semen, urine, and blood samples. They were requested to abstain from ejaculation for at least 3 days, but no more than 10 days prior to their visit. Semen was collected on site by masturbation without the use of lubricants into a sterile polypropylene collection container (Cardinal Health, Dublin, OH). The *World Health Organization (WHO) Laboratory Manual for the Examination and Processing of Human Semen* 5th edition was referenced for normal values [28]. Semen was analyzed for standard clinical parameters after liquefaction, no later than 60 min from collection. After completion of these clinical sperm analyses, the samples were subjected to two-step ISolate-gradient centrifugation (Irvine Scientific) to select a motile population enriched in normal morphology. This colloidal silica gradient, consisting of a 90 % lower layer and 50 % upper layer, is prepared by sequentially adding 1.5 ml of each to a 15 ml polystyrene conical tube. The sperm sample was pipetted on top of the upper layer and centrifuged at 200×*g* for 15 mins. The gradient solution was removed and the pelleted sperm frozen and stored at −80 °C for subsequent DNA methylation analyses.

### DNA methylation analysis

The differentially methylated regions (DMRs) associated with the following genes were examined: The two DMRs analyzed for the *DLK1/MEG3* imprinted domain consisted of the *MEG3-*IG DMR (4 CpG sites) and *MEG3* DMR (8 CpG sites) (chr 14q32.2). The region upstream from the *IGF2* imprinted gene promoters included three CpG dinucleotides (chr 11p15.5). The DMR for *H19* included four CpG sites (chr 11p15.5). The following gene promotor DMRs were also tested: *GRB10* (6 CpG sites) (chr 7p12.2), *NDN* (6 CpG sites) (chr 15q11.2), *NNAT* (3 CpG sites) (chr 20q.11.2), *PLAGL1* (6 CpG sites) (chr 6q24), *SGCE/PEG10* (6 CpG sites) (chr 7q21.3), *SNRPN* (4 CpG sites) (chr 15q11.2), *PEG1/MEST* (4 CpG sites) (chr 7q21.3), and *PEG3* (10 CpG sites) (chr 19q13.43) [29, 30].

Genomic DNA was extracted from sperm samples using Puregene Reagents (Qiagen; Valencia, CA). Genomic DNA (800 μg) was treated with sodium bisulfite using the Zymo EZ DNA Methylation Kit (Zymo Research; Irvine, CA), converting unmethylated cytosines to uracils. Methylated cytosine is unaltered by this treatment. After bisulfite treatment, DNA (~20 ng) was amplified by PCR using the PyroMark PCR Kit (Qiagen) with 1.5 mM MgCl_2_, 0.12 μM each of forward and reverse PCR primers, and 2.5 μl of CoralLoad concentrate (Qiagen). Primers used for each region of interest were designed to anneal to sequences with non-methylated CpG sites. Primer sequences used for most DMRs can be found in previous studies [29, 30]. This study also investigated *PLAGL1*, *NDN*, *SNRPN*, and *GRB10.* Primer information for these genes can be found in Additional file 1: Table S1. The 5′ end of one primer from each pair was conjugated to biotin, allowing for retention of one DNA strand through annealing to streptavidin beads. The Pyrosequencing Work Station was used to isolate the single strand, and then pyrosequencing was completed using the PyroMark Q96 MD pyrosequencing instrument (Qiagen). Bisulfite pyrosequencing assays used to measure methylation levels at most CpG sites have also been published previously [29, 30]. For genes with previously unpublished assay validation data, Additional file 2: Figure S1 shows that experimentally determined methylation percentage measures correlate closely with the known methylation of validation samples. The sensitivity of pyrosequencing to detect changes at very low levels of DNA methylation was performed using the same technique for preparing defined mixtures of DNA as we have previously published [29, 30]. We tested the ability of pyrosequencing to detect differences in methylation below 5 %, using defined mixtures of fully methylated and unmethylated DNAs hand pipetted in 0.5 % increasing increments of methylated DNA from 0 to 5 %. As shown in Additional file 3: Figure S2, pyrosequencing was able to detect 0.5 % differences across this very low-level range of DNA methylation.

### Statistical analysis

Variables were defined as follows: age (18–24, 25–29, 30–35), highest degree of education (high school, some college/college, or graduate degree), marital status (single, married/living with partner, or divorced/widow), fathered children (yes or no), number of hours of intensive exercise per week (less than 4 or 4 h or more), number of hours spent watching TV, seated or inactive per week (less than 4 h per week or 4 h or more), sperm concentration (less than 1.5 × 10^7^ or 1.5 × 10^7^ spermatozoa/ml or more), sperm motility (less than 40 or 40 % or more), total sperm count or TMC (less than or equal to 3.9 × 10^7^ or more than 3.9 × 10^7^), and fertility clinic patient status (yes or no). Chi-square tests were used to compare overweight/obesity status of the participants within various subgroups. If the number of participants was small (<5), Fisher’s exact test was used. Body mass index (BMI) was calculated from height and weight measured by our study nurse the day of the participant’s visit. In accordance with WHO guidelines, the following categories were defined: normal weight (18.5 kg/m^2^ ≤ BMI < 25 kg/m^2^), overweight (25 kg/m^2^ ≤ BMI < 30 kg/m^2^), and obese (BMI ≥ 30 kg/m^2^). For the purposes of this analysis, individuals with BMIs over 25 were categorized as “overweight/obese”.

The mean DNA methylation percentages at each CpG site and the mean for the overall DMR in normal weight men versus overweight/obese men were compared using a Mann-Whitney *U* test. These analyses were extended by using multivariate regression models, adjusting for potential confounding factors. Potential confounders were selected based on known or observed associations with DNA methylation and with obesity; in the final, main analysis, we controlled for age and patient status at the fertility clinic as confounders. Sensitivity analyses using subgroups of our participants and various multivariate analyses, with and without our variables, were repeated and compared. All analyses were based on the available laboratory data for each CpG site at the DMRs. Statistical analyses were conducted in SAS 9.4 (SAS Institute Inc., Cary, NC, USA).

### Ethics

The TIEGER study was performed with the approval of the Duke University Institutional Review Board (reference number: Pro00036645). Informed consent was obtained from all participants for the use of their biological specimens and questionnaire data.

## Results

### Characteristics of study participants

The distributions of the characteristics of study participants by category of BMI are shown in Table 1. Twenty-three men were categorized as overweight/obese, representing 33.3 % of our study population. More specifically, 11 men were overweight (25 ≤ BMI < 30) and 12 men were obese (BMI ≥ 30). Having a BMI of 25 or more was strongly associated with increased age (*p* = 0.001). Most men in the overweight/obese category were older than 25 years (86.9 %), while less than half (45.7 %) in the normal BMI category were older than 25. Marital status was also strongly associated with overweight/obesity. Most overweight/obese men were married (73.9 %), while most men of normal weight were single (68.9 %) (*p* = 0.0003). Increased age was also associated with being married (*p* < 0.001). Increased age and overweight/obesity status were both strongly associated with being a patient at the fertility clinic (both *p* values <0.001). We examined if the clinical sperm characteristics were associated with overweight/obesity status and found no significant associations. Overweight/obesity status and aberrant sperm motility indicated a potential relationship, but this was not significant: motility was abnormal (less than 40 % motile) in 30.4 % of the overweight and obese men, while only 14.0 % of the normal weight participants had an abnormal sperm motility count (*p* = 0.11).

**Table 1:** Socio-demographic data of male volunteers

TIEGER participants		Normal weight *n* = 46		Overweight or obese *n* = 23	
		*n*	%	*n*	%
Age	18–24 years	25	54.3	3	13.0
	25–29 years	12	26.1	7	30.4
	30–35 years	9	19.6	13	56.5
	*p* value	0.001			
Highest degree of education	High school	6	13.3	1	4.3
	Some college or college degree	27	60.0	15	65.2
	Graduate	12	26.7	7	30.4
	*p* value	0.59			
Marital status	Single	31	68.9	5	21.7
	Married/living with partner	14	31.1	17	73.9
	Divorced/widow	0	0	1	4.3
	*p* value	0.0003			
Having children^a^	No	41	89.1	19	82.6
	Yes	5	10.9	4	17.4
	*p* value	0.47			
Watching TV (inactive/seated)	Less than 4 h per week	17	37.0	4	17.4
	4 h or more per week	29	63.0	19	82.6
	*p* value	0.16			
Exercise (heavy/sweating)	Less than 2 h per week	16	34.8	11	47.8
	2 h or more per week	30	65.2	12	52.2
	*p* value	0.29			
Sperm concentration	<1.5 × 10^7^	3	7.0	1	4.3
	≥1.5 × 10^7^	40	93.0	22	95.7
	*p* value	0.67			
Sperm total motility count	≤3.9 × 10^7^	8	18.6	4	17.4
	>3.9 × 10^7^	35	81.4	19	82.6
	*p* value	0.90			
Sperm motility	<40 %	6	14.0	7	30.4
	≥40 %	37	86.0	16	69.6
	*p* value	0.11			
Patient at Fertility clinic	No	40	87.0	9	39.1
	Yes	6	13.0	14	60.9
	*p* value	<0.001			

If the sum was not 46 (normal BMI) or 23 (overweight/obese), respectively, data were missing and percentage was calculated on known data. Chi-square test was applied, except if *n* ≤ 5, in this case Fisher exact test is used

^a^Having children: only one was conceived through ART; this was in the category of men with normal weight

### Methylation at imprinted genes in sperm DNA of TIEGER participants

Methylation profiles of sperm DNA from men with normal weight are presented in Table 2. As anticipated, the following gene DMRs were nearly 100 % methylated (range, 80.0 to 93.9 %): *MEG3-IG*, *H19*, and *IGF2*, consistent with the known paternal origin of methylation for these regions*.* Other gene DMRs studied were nearly completely unmethylated (range, 1.6 to 4.1 % methylated), consistent with the known maternal origin of methylation for these regions and the requirement that these regions are demethylated in sperm prior to conception. One exception was the DMR for *MEG3*, which was unmethylated (approximately 0 %) in sperm, despite being a known paternally methylated DMR. However, this DMR is known to acquire methylation post-fertilization in mouse [31]; thus, this methylation pattern is still consistent with a paternal origin.

**Table 2:** Methylation profiles in sperm from men with normal weight at the DMRs of imprinted genes

	Methylation % in sperm from men with normal BMI																							
	*MEG3-IG*		*MEG3*		*PEG1/MEST*		*IGF2*		*H19*		*GRB10*		*NNAT*		*NDN*		*SNRPN*		*SGCE/PEG10*		*PEG3*		*PLAGL1*	
	%	SE	%	SE	%	SE	%	SE	%	SE	%	SE	%	SE	%	SE	%	SE	%	SE	%	SE	%	SE
CpG 1			*0.84*	0.38	*0.65*	0.11	*91.80*	0.25	*95.28*	0.40	*0.87*	0.19	*0.61*	0.19	*1.90*	0.32	*2.53*	0.18	*3.48*	0.57	*1.50*	0.22	*4.03*	0.89
CpG 2	*79.02*	0.48	*2.68*	0.53	*1.20*	0.08	*92.90*	0.36	*66.71*	0.93	*2.81*	0.17	*1.90*	0.25	*1.26*	0.34	*1.59*	0.16	*1.91*	0.58	*1.62*	0.25	*3.90*	0.91
CpG 3	*73.99*	0.36	*1.83*	0.56	*2.06*	0.14	*96.99*	0.37	*92.79*	0.34	*1.32*	0.17	*2.99*	0.28	*1.27*	0.21	*1.86*	0.19	*3.69*	0.61	*1.58*	0.24	*4.57*	0.82
CpG 4	*85.85*	0.46	*3.10*	0.51	*2.35*	0.19			*95.29*	0.31	*1.22*	0.18			*1.54*	0.31	*0.74*	0.15	*4.25*	0.56	*1.19*	0.21	*4.20*	0.91
CpG 5	*80.01*	0.37	*1.71*	0.56							*2.75*	0.16			*1.58*	0.30			*2.63*	0.59	*1.70*	0.27	*3.99*	0.86
CpG 6			1.89	0.56							*2.14*	0.16			*2.10*	0.25			*4.44*	0.63	*1.05*	0.25	*3.32*	0.77
CpG 7			2.52	0.53																	*1.91*	0.24		
CpG 8			2.21	0.52																	*0.92*	0.22		
CpG 9																					*2.23*	0.24		
CpG 10																					*1.97*	0.21		
Mean	*79.72*	0.33	*2.10*	0.51	*1.56*	0.08	*93.90*	0.28	*87.51*	0.34	*1.85*	0.16	*1.83*	0.26	*1.61*	0.28	*1.65*	0.16	*3.40*	0.58	*1.57*	0.23	*4.00*	0.84

Results shown here describe methylation profiles for normal weight men (BMI < 25). The DNA methylation percentages are presented for each DMR, by the individual CpG site studied. The calculated mean for each DMR is shown on the bottom line

### Methylation levels at imprinted genes in sperm of overweight/obese men versus normal weight men

**Fig. 1:**
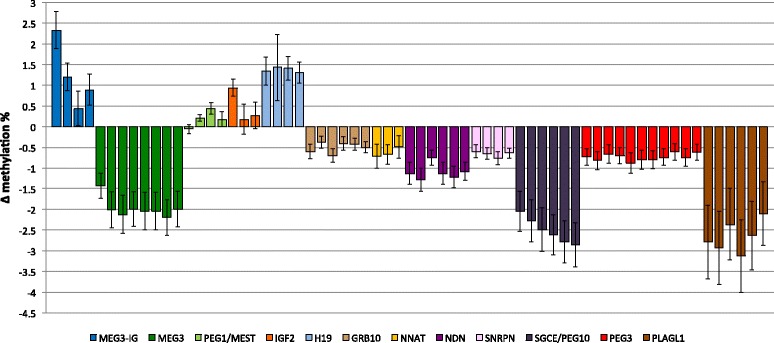
Methylation differences in sperm from overweight/obese men versus normal weight men at imprinted genes. Differences in methylation percentages between overweight/obese men and men of normal weight are shown by CpG site for each DMR studied, adjusted for age and patient status at the Duke Fertility Center. *Bars* represent SE

At least two of the 12 DMRs studied in sperm were differentially methylated by obesity or overweight status. Sperm of overweight or obese men had a significantly higher (2.8 %) DNA methylation level than sperm of normal weight men (*p* < 0.001) at the first CpG-site of the *MEG3-IG* DMR. The *H19* DMR showed a mean methylation difference of +4.1 % (*p* = 0.012) and +1.2 % (*p* = 0.020) at CpG2 and CpG3, respectively (Additional file 4: Table S2). After adjusting for age and patient status in our multivariate analyses (Table 3), overweight/obese status was associated with higher DNA methylation at the first CpG site examined of *MEG3-IG* (β = +2.33; SE = 0.89; *p* = 0.01) and at CpG1 of *IGF2* (β = +0.94; SE = 0.41; *p* = 0.02). An indication for an association was also present at CpG3 of *PEG1/MEST* (β = +0.44; SE = 0.27; *p* = 0.11). At three out of four CpG sites of *H19*, we also found significantly higher DNA methylation in overweight/obese participants at CpG1 (β = +1.34, SE = 0.68; *p* = 0.05), CpG3 (β = +1.41, SE = 0.58; *p* = 0.02), and CpG4 (β = +1.31, SE = 0.51; *p* = 0.01). Lower DNA methylation percentages were measured at all CpG sites of *MEG3*, and the mean methylation difference between obese/overweight men and normal BMI men was −1.99 (SE = 0.84; *p* = 0.02). All CpG sites of *NNAT*, *PEG3*, and *PLAGL1* showed slightly lower DNA methylation levels if men were overweight/obese, but results were not significant. All CpG sites of *NDN*, *SNRPN*, and *SGCE/PEG10* showed significantly lower DNA methylation if men were overweight/obese. The mean β-coefficients were −1.10 (SE = 0.47; *p* = 0.02), −0.65 (SE = 0.27; *p* = 0.02), and −2.51 (SE = 1.01; *p* = 0.01), respectively. At *GRB10*, DNA methylation was also slightly lower at all sites, but only CpG3 reached significance (β = −0.70, SE = 0.31; *p* = 0.03). Figure 1 represents the adjusted DNA methylation changes for overweight or obese men.

**Table 3:** Multivariate analyses: DNA methylation at the DMRs of imprinted genes in sperm cells of obese and overweight volunteers versus normal weight men

	Imprinted genes																	
CpG sites	*MEG3-IG*			*MEG3*			*PEG1/MEST*			*IGF2*			*H19*			*GRB10*		
	%	SE	*p*	%	SE	*p*	%	SE	*p*	%	SE	*p*	%	SE	*p*	%	SE	*p*
CpG 1				*−1.43*	0.62	0.02	*−0.06*	0.21	0.76	*+0.94*	0.41	0.02	*+1.34*	0.68	0.05	*−0.60*	0.35	0.09
CpG 2	*+2.33*	0.89	0.01	*−2.01*	0.88	0.03	*+0.21*	0.16	0.19	*+0.18*	0.72	0.81	*+1.43*	1.61	0.38	*−0.38*	0.29	0.20
CpG 3	*+1.20*	0.66	0.08	*−2.12*	0.92	0.02	*+0.44*	0.27	0.11	*+0.27*	0.66	0.69	*+1.41*	0.58	0.02	*−0.70*	0.31	0.03
CpG 4	*+0.44*	0.83	0.59	*−1.99*	0.84	0.02	*+0.18*	0.38	0.64				*+1.31*	0.51	0.01	*−0.41*	0.32	0.21
CpG 5	*+0.89*	0.76	0.24	*−2.04*	0.91	0.03										*−0.42*	0.29	0.15
CpG 6				*−2.04*	0.91	0.03										*−0.50*	0.28	0.08
CpG 7				*−2.19*	0.86	0.01												
CpG 8				*−1.99*	0.86	0.02												
CpG 9																		
CpG 10																		
Mean	*+1.22*	0.59	0.04	*−1.99*	0.84	0.02	*+0.19*	0.17	0.26	*+0.46*	0.52	0.38	*+1.37*	0.62	0.03	*−0.50*	0.29	0.09
CpG site	*NNAT*			*NDN*			*SNRPN*			*SGCE/PEG10*			*PEG3*			*PLAGL1*		
	%	SE	*p*	%	SE	*p*	%	SE	*p*	%	SE	*p*	%	SE	*p*	%	SE	*p*
CpG 1	*−0.71*	0.58	0.22	*−1.13*	0.53	0.04	*−0.60*	0.31	0.05	*−2.04*	0.98	0.04	*−0.73*	0.40	0.07	*−2.79*	1.78	0.12
CpG 2	*−0.67*	0.47	0.16	*−1.28*	0.56	0.03	*−0.65*	0.28	0.02	*−2.27*	1.02	0.03	*−0.82*	0.44	0.07	*−2.93*	1.76	0.10
CpG 3	*−0.49*	0.55	0.37	*−0.75*	0.36	0.04	*−0.76*	0.33	0.02	*−2.49*	1.05	0.02	*−0.66*	0.45	0.14	*−2.36*	1.72	0.17
CpG 4				*−1.13*	0.53	0.04	*−0.64*	0.25	0.01	*−2.61*	0.97	0.01	*−0.70*	0.38	0.07	*−3.13*	1.74	0.08
CpG 5				*−1.22*	0.52	0.02				*−2.78*	1.02	0.01	*−0.88*	0.50	0.08	*−2.63*	1.67	0.12
CpG 6				*−1.08*	0.44	0.02				*−2.85*	1.07	0.01	*−0.80*	0.45	0.08	*−2.10*	1.54	0.18
CpG 7													*−0.74*	0.43	0.09			
CpG 8													*−0.61*	0.39	0.12			
CpG 9													*−0.75*	0.43	0.09			
CpG 10													*−0.62*	0.39	0.12			
Mean	*−0.62*	0.50	0.22	*−1.10*	0.47	0.02	*−0.65*	0.27	0.02	*−2.51*	1.01	0.01	*−0.73*	0.42	0.09	*−2.66*	1.67	0.12

The model shown adjusts for age (as a continuous variable) and being a patient (yes/no) at the fertility clinic as possible confounding variables. Percentages for each CpG site represent the deviation of methylation percentage for overweight/obese men (BMI ≥ 25) from the methylation percentage of normal weight men (BMI < 25). These deviations were also averaged over all CpG sites associated with a particular imprinted gene (shown on the bottom line).

In order to assess the potential for selection bias by age, these analyses were repeated, restricting the study sample to 41 men aged 25 to 35. In this age range, the age distribution by BMI category was similar. Although this reduction in sample size decreased precision of our estimates, the overall findings did not change (data not shown), suggesting that the association between overweight or obesity and DNA methylation at these regions is not unduly influenced by age.

## Discussion

Most studies on epigenetic effects of obesity are conducted in mother-child dyads, and the effects of the paternal germ line are rarely examined. We undertook an analysis of the association between paternal overweight/obesity status and sperm methylation percentage at the regulatory regions of imprinted genes *MEG3-IG*, *MEG3*, *PEG1/MEST*, *IGF2, H19,GRB10*, *NNAT*, * NDN*, *SNRPN*, *SGCE/PEG10*, *PLAGL1*, and *PEG3*. We found significant differences in DNA methylation patterns between overweight/obese men and men of normal BMI at multiple DMRs. An important finding was that a fraction of the mature haploid spermatozoa in each sample had not acquired their fully methylated or unmethylated status at imprinted DMRs, as is expected for paternally or maternally methylated DMRs, respectively. Intriguingly, we found that after adjusting for potential confounding by age and patient status, DMRs that should be unmethylated in sperm (maternally methylated DMRs) were closer to 0 % methylation if men were overweight or obese. These genes included *GRB10*, *NDN*, *SNRPN*, and *SGCE/PEG10*. In addition, overweight/obese men had a higher fraction of sperm with complete DNA methylation at regions where paternal methylation (closer to 100 %) is expected, as compared to normal weight men. This was the case at the *IGF2* and *H19* DMRs. The *MEG3-IG* and *MEG3* DMRs are separated by about 20 kb in humans and are unusual in terms of establishment of paternal imprint marks. According to studies in mice [31], methylation at these two DMRs is acquired at different developmental time points: the *MEG3-IG* DMR methylation is established during maturation of sperm, while the *MEG3* DMR acquires methylation only after fertilization. Both regions play an essential role in expression regulation of *MEGs* and *PEGs* in the human body [32]. In semen of overweight/obese men, we observed that a higher fraction of sperm cells were fully methylated at the DMR of *MEG3-IG* as compared to semen from normal weight men, while in the same subgroup of overweight/obese men, DNA methylation at the DMR of *MEG3* was closer to 0 %. Hence, in overweight or obese men, both regions had a tendency to be closer to the theoretically expected levels for methylation in these regions.

Interestingly, analyses in the Newborn Epigenetic STudy (NEST) cohort showed that newborns of obese fathers had comparable methylation patterns at some imprinted gene DMRs. A matching positive trend between high BMI and methylation at *H19* and *MEG3-IG* DMRs was observed in the NEST cohort, but results did not reach significance. Similar paradoxical results regarding expected theoretical levels of methylation were found. Lower DNA methylation was seen at DMRs of genes expected to be unmethylated on the paternal allele (e.g*. PEG3*, *NNAT*, and *PEG1/MEST*) [25]. In the current study, sperm from overweight/obese men also had a lower, albeit not significant methylation profile at *PEG3* and *NNAT.* Although the results—comparing the same DMRs in this study to those children born to obese fathers in the NEST cohort study—were not all significant, the overall directions of associations are consistent with the notion that methylation differences detected in the sperm of overweight/obese men in this study could persist in their offspring. Taken together, the results of the NEST and TIEGER studies suggest that linkages between sperm and offspring epigenetic modifications merit further investigation. Human data on paternal obesity and its potential effects on sperm and offspring epigenetics are limited. To our knowledge, only one recent study investigated a potential effect of obesity on the sperm epigenome. Donkin et al. performed a comprehensive epigenetic mapping of sperm from obese versus normal weight men [33]. Despite their limited sample size (10 obese men), potential for selection bias, and the fact that different CpG sites were examined, the Donkin et al. study corroborates our findings that methylation differences exist in the sperm methylome for obese men versus lean men. Some studies indicate that the effects of epigenetic modifications through sperm can be substantial for the offspring. Feinberg et al. found that within a group of men who had already fathered a child with autism, differences in methylation in the sperm genome were present at genes associated with autism spectrum disorder phenotypes. These results suggest that epigenetic differences in sperm may have profound offspring phenotypic consequences [34]. Methylation profiles of sperm also may impact offspring viability. In a study of patients undergoing IVF, sperm DNA methylation patterns were found to be predictive of IVF embryo quality [35]. In animal models, deregulated methylation at imprinted genes has been observed in sperm from obese parents, which was associated with altered developmental capacity of the embryo and reduced pluripotency and metabolic function [36, 37]. Although the relationship between BMI and clinical sperm parameters remains controversial in humans, there is growing evidence that male obesity is related to decreased fertility [38], which may be related to impaired DNA methylation in sperm [39]. Epigenetic effects from obese mothers on their son’s sperm have also been reported. Ge et al. investigated DNA methylation patterns in a mouse model at imprinted genes in sperm of offspring born to obese and/or diabetic dams. DNA methylation was increased at DMRs of the *H19* and *Peg3* genes [40]. In the current study, similar regions are differentially methylated by obesity status. However, only DNA methylation at *H19* was significant in men. From these data, it can be concluded that molecular mechanisms, hormonal imbalances, diet, or other obesity-related factors could be the cause of epigenetic changes in sperm. Several animal studies have demonstrated that the sperm epigenome may be responsive to dietary factors. Effects were measured through negative pregnancy outcomes, altered gene expression patterns in the offspring, and/or diminished reproductive health in subsequent generations [10, 11, 22]. However, a recent study in mice by Shea et al. reported conflicting results demonstrating that the sperm methylome was much more strongly correlated with epigenetic variations occurring randomly or due to other unknown factors rather than diet [41]. Their findings suggest that obesity and body composition are more important than diet composition, or that diet may have an influence via modified body composition. For instance, obesity is associated with elevated estrogen levels, and results from animal studies suggest that increased exposure to estrogen in the testes may lead to abnormal methylation patterns, providing a possible mechanism for how body fat can impact DNA methylation [38, 42]. Further investigation is needed to disentangle the independent contributions of diet versus body composition or its metabolic/hormonal status in regard to sperm epigenetics. Another environmental factor that could also induce epigenetic modifications in sperm DNA is testis hyperthermia in obese subjects. Animal data provides evidence that heat can perturb the dynamics of DNA methylation programming in the paternal genome; this was shown in bulls by Rahman et al. [43]. A recent report in humans provides evidence that the state of varicocele, a condition related to heat affecting sperm, is related to DNA methylation in spermatozoa [44]. Hence, we cannot exclude the possibility that obesity-related genital heat could have influenced our results.

Other exposures, such as to the endocrine-disrupting factor BPA or its metabolites, may also be involved. These environmental factors could play a role in influencing both weight gain and spermatogenesis, and thus, it cannot be completely ruled out that both increased BMI and methylation changes are parallel results of environmental exposures [45]. Future studies should strive to elucidate the underlying mechanisms by which overweight/obesity may affect methylation differences, and if a third factor, such as an environmental exposure or nutritional profiles, may be influencing both outcomes.

The methylation changes we found in this study were subtle. However, these small changes in sperm may still have an impact on offspring phenotypes. Murphy et al. reported that a 1 % change in methylation at the *IGF2* DMR leads to either a doubling or halving of *IGF2* transcription, depending on the direction of methylation. This degree of change is equivalent to complete loss of imprinting, which is particularly significant because this loss of imprinting is often observed in cancers [46]. The modest methylation changes in sperm associated with obesity found here are also consistent with methylation changes induced in humans by other early-life environmental exposures and lifestyle factors, including prenatal exposure to famine [47], maternal nutrition supplementation [48], use of medicines during pregnancy [49, 50], and maternal exposure to cadmium [51] and lead (in press by Nye and Hoyo in Environmental Epigenetics). Large changes in methylation may in fact be less meaningful in regard to intergenerational impact because significant methylation aberrations may not result in viable offspring.

Although others have done a similar analysis in smaller populations [33], a possible weakness of this study remains the limited statistical power to detect significant differences. Furthermore, one third of our population was recruited at the Duke Fertility Center. Although we adjusted for Fertility Center patient status, there may be residual confounding resulting from inherent differences in characteristics of sperm DNA between (obese) men with potential fertility problems attending the clinic and those not attending the clinic. Hence, our results on sperm epigenetic differences may partly reflect the effects in patients seeking care at a fertility clinic, rather than the general population. Additionally, we focused on self-reported Caucasian men only, while North Carolina also includes a substantial population from other ethnicities or origins, with a higher prevalence of obesity, e.g., African Americans [52]. Given the increasing prevalence of obesity in males, our provocative findings require replication in a larger multiethnic study.

Strengths of our study include that our study population was comprised of men of reproductive age. We further did not sub select our obese and non-obese groups by any criteria. No exclusions were made based on health conditions (other than those related to fertility), as was done in the Donkin et al. study [33]. We focused on a group of regulatory elements that are established during spermatogenesis (or early post-fertilization in the case of the *MEG3* DMR) and that are critical in the programming of the correct expression patterns of imprinted genes. Because imprinted genes are generally spared the global reprograming post-fertilization, methylation patterns established during spermatogenesis are persistent, and thus transferred to the offspring.

A remarkable finding in our descriptive analysis was the fact that the epigenetic imprinting is not as “complete” as theoretically expected, and that some paternally imprinted genes (e.g., *MEG3*) are not fully methylated until after fertilization. There are currently no comparable epigenetic studies on human (maturing) sperm in the literature for us to compare our results against, highlighting the lack of research on gametic epigenetic profiles at various stages of maturation and development. Hence, we can only suggest hypotheses for why men with normal BMI have a larger fraction of epigenetically “incomplete” sperm cells. One possible explanation is that a larger deviation from the expected methylation pattern in sperm reflects normal epigenetic variation at the DMRs on a population level, allowing for adaptive phenotypic plasticity in response to environmental changes [5]. A chronic exposure to an obesity-related microenvironment in the testes during spermatogenesis may alter the sperm epigenome. If this exposure persists through generations, it may influence long-term evolutionary trends [12]. Despite the observed associations between paternal obesity, epigenetic modification, and offspring phenotype, the timing and nature of paternal obesity’s influence on sperm and consequently offspring methylation profiles is potentially important. Persistent changes in epigenetic profiles may vary if the exposure starts during early development of the male gametes, before puberty, or at later age [3]. Overall, our results point to a need for investigation of the impact of the environment on the human sperm epigenome, as well as its normal variation.

## Conclusions

The current study focused exclusively on the programming of imprint marks during male gametogenesis. Our results indicate that obesity, or its related factors, can change epigenetic programming in a small fraction of sperm cells. The contribution of paternal obesity towards fetal and later adult development is especially relevant due to the global obesity trend and merits further exploration. Fortunately, studies have also indicated that paternal effects on future offspring can be prevented by weight loss and exercise [53]. Epigenetics may provide the key for the intergenerational influences of obesity and other lifestyle factors of both mothers and fathers on offspring health. If paternal obesity in fact does have an effect on offspring later health and development, then the need for lifestyle change is even more urgent.

## Additional files

Additional file 1: Table S1. Primer table. (DOCX 48 kb) Additional file 2: Figure S1. Pyrosequencing validation of the *PLAGL1*, *GRB10*, *NDN*, and *SNRPN* assays. Defined mixtures of Qiagen Epitect Bisulfite Modified control DNAs (0, 25, 50, 75, and 100 % methylated; *x*-axis) were analyzed by pyrosequencing, with the actual percent methylation measured shown on the *y*-axis. Error bars indicate the standard deviation for triplicate measures. (DOCX 161 kb) Additional file 3: Figure S2. Pyrosequencing validation of the *IGF2* assay at very low methylation levels. Defined mixtures of Qiagen Epitect Bisulfite Modified control DNAs (from 0 to 5 % methylation, in 0.5 % increments) were analyzed by pyrosequencing, with the actual percent methylation measured shown on the *y*-axis. Error bars indicate standard deviation for triplicate measures. (DOCX 190 kb) Additional file 4: Table S2. Differences between DNA methylation in sperm from men with normal weight versus overweight/obese men at the DMRs of imprinted genes. (DOCX 53 kb)

## Abbreviations

BMI: body mass index
DMRs: differentially methylated regions
*GRB10*: growth factor receptor-bound protein 10
*IGF2*: insulin-like Growth Factor 2
*MEG3*: maternally expressed gene 3
*NDN*: necdin
NEST: Newborn Epigenetics STudy
*NNAT*: neuronatin
*PEG1*/*MEST*: paternally expressed gene 1/mesoderm specific transcript
*PEG3*: paternally expressed gene 3
*PLAGL1*: pleiomorphic adenoma gene-like 1
*SGCE*/*PEG10*: epsilon sarcoglycan/paternally expressed gene 10
*SNRPN*: small nuclear ribonucleoprotein polypeptide N
TIEGER: The Influence of the Environment on Gametic Epigenetic Reprogramming
