# Supplementary material for: Obesity-related DNA methylation at imprinted genes in human sperm: Results from the TIEGER study
Source: Clin Epigenetics. 2016 May 6;8:51. doi: 10.1186/s13148-016-0217-2 (PMC4859994; doi:10.1186/s13148-016-0217-2)
Supplement: Additional file 1: Table S1. — Primer table. (DOCX 48 kb) [file 13148_2016_217_MOESM1_ESM.docx]

**Suppl. Table 1. Primer table**

| DMR (Chr) | Forward Primer | Reverse Primer | Sequencing Primer |
| --- | --- | --- | --- |
| *NDN* (15q11.2) | ATAGGTTTTTTGTTTATGATTTTGAGT | *CAACTCCTCAATAATAAACTTCC | AGTTTTATTTAAGTGAAGGG |
| *GRB10* (7p12.2) | *AGGAGGTAGTGGAGGGAATAAG | CCCAAAACCAAACCCATATA | CAAAACCAAACCCATATA |
| *SNRPN (*15q11.2) | AAAGTTTTTTGTTTTGGAGAATTAGATT | *ATATACCCACCTCCACCCATAT | TTTGTTTAGATGGTATTT |
| *PLAGL1* (6q24) | GTAGGgtaggtgtttgggtgTt | *gaggagggtgtgTTtttgTCG | gtaggtgtttgggtgTt |

*Denotes biotinated primer
