# Supplementary material for: Obesity-related DNA methylation at imprinted genes in human sperm: Results from the TIEGER study
Source: Clin Epigenetics. 2016 May 6;8:51. doi: 10.1186/s13148-016-0217-2 (PMC4859994; doi:10.1186/s13148-016-0217-2)
Supplement: Additional file 2: Figure S1. — Pyrosequencing validation of the PLAGL1, GRB10, NDN, and SNRPN assays. Defined mixtures of Qiagen Epitect Bisulfite Modified control DNAs (0, 25, 50, 75, and 100 % methylated; x-axis) were analyzed by pyrosequencing, with the actual percent methylation measured shown on the y-axis. Error bars indicate the standard deviation for triplicate measures. (DOCX 161 kb) [file 13148_2016_217_MOESM2_ESM.docx]

**Suppl. Figure 1. Pyrosequencing validation of the *PLAGL1, GRB10, NDN* and *SNRPN* assay.**
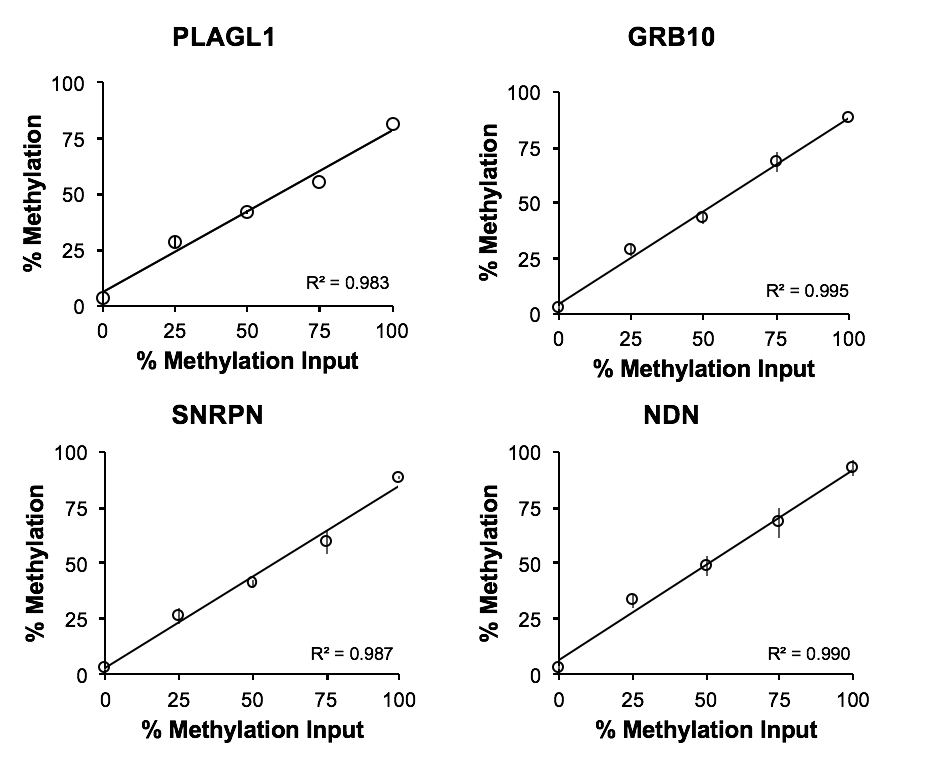


Defined mixtures of Qiagen Epitect Bisulfite Modified control DNAs (0%, 25%, 50%, 75% and 100% methylated; x-axis) were analyzed by pyrosequencing, with the actual percent methylation measured shown on the y-axis. Error bars indicate the standard deviation for triplicate measures.
