# Supplementary material for: Obesity-related DNA methylation at imprinted genes in human sperm: Results from the TIEGER study
Source: Clin Epigenetics. 2016 May 6;8:51. doi: 10.1186/s13148-016-0217-2 (PMC4859994; doi:10.1186/s13148-016-0217-2)
Supplement: Additional file 3: Figure S2. — Pyrosequencing validation of the IGF2 assay at very low methylation levels. Defined mixtures of Qiagen Epitect Bisulfite Modified control DNAs (from 0 to 5 % methylation, in 0.5 % increments) were analyzed by pyrosequencing, with the actual percent methylation measured shown on the y-axis. Error bars indicate standard deviation for triplicate measures. (DOCX 190 kb) [file 13148_2016_217_MOESM3_ESM.docx]

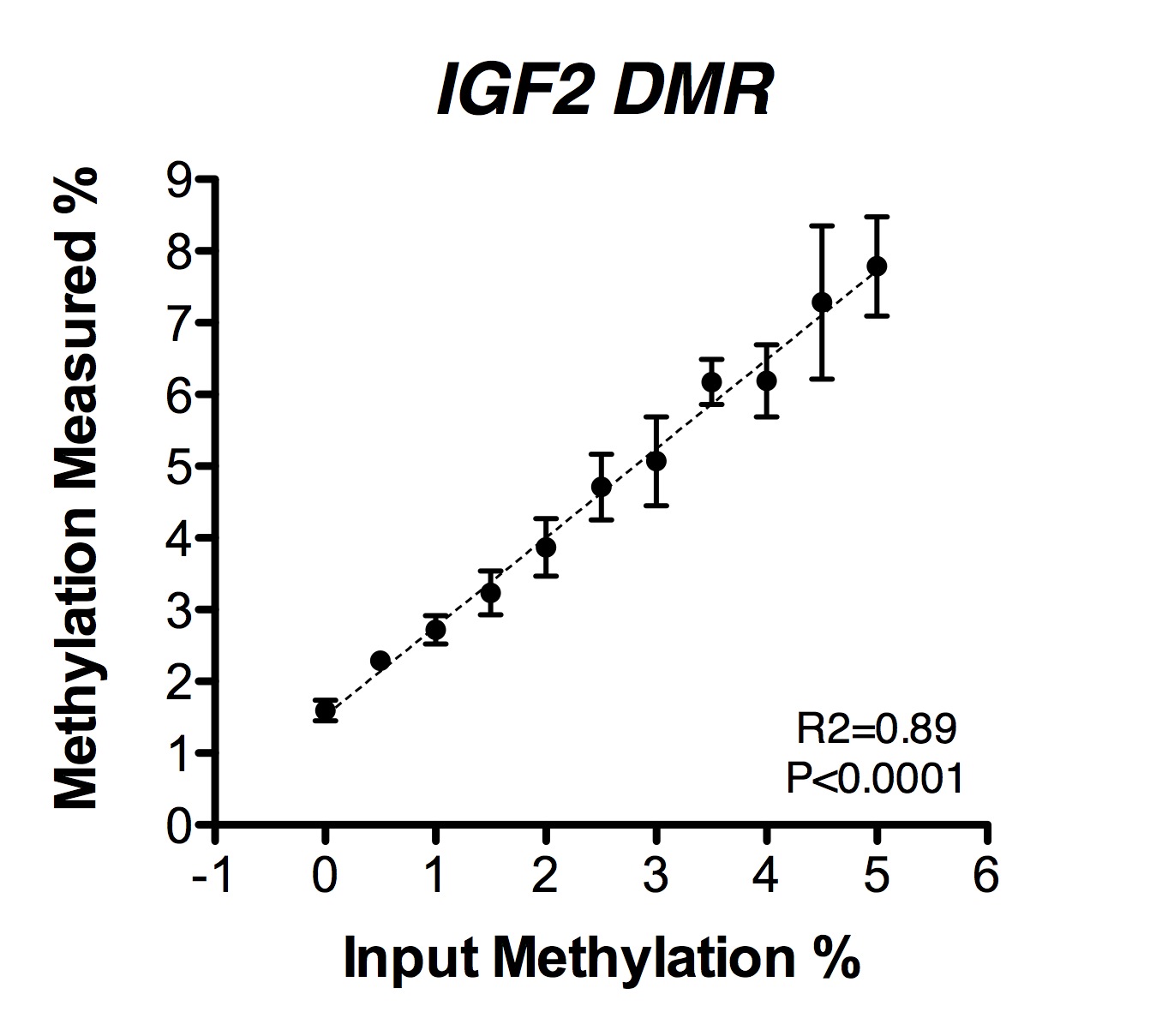
**Suppl. Figure 2. Pyrosequencing validation of the *IGF2* assay at very low detection levels.**

Defined mixtures of Qiagen Epitect Bisulfite Modified control DNAs (from 0% to 5% methylation, in 0.5% increments) were analyzed by pyrosequencing, with the actual percent methylation measured shown on the y-axis. Error bars indicate standard deviation for triplicate measures.
