# Supplementary material for: Obesity-related DNA methylation at imprinted genes in human sperm: Results from the TIEGER study
Source: Clin Epigenetics. 2016 May 6;8:51. doi: 10.1186/s13148-016-0217-2 (PMC4859994; doi:10.1186/s13148-016-0217-2)
Supplement: Additional file 4: Table S2. — Differences between DNA methylation in sperm from men with normal weight versus overweight/obese men at the DMRs of imprinted genes. (DOCX 53 kb) [file 13148_2016_217_MOESM4_ESM.docx]

**Suppl. Table 2. Differences between DNA methylation in sperm from men with normal weight *versus* overweight/obese men at the DMRs of imprinted genes.**

|  | **mean methylation % (SD)** | | **p-value** |
| --- | --- | --- | --- |
|  | **normal weight** | **overweight//obese** |  |
| **H19_CpG2** | 66.7 (6.0) | 70.8 (5.4) | 0.012 |
| **H19_CpG3** | 92.8 (2.2) | 94.0 (0.8) | 0.020 |
| **MEG3-IG_CpG2** | 79.0 (3.2) | 81.8 (2.2) | <0.001 |

The DNA methylation mean percentages are presented for all CpG sites that were significantly different by BMI status (p<0.05). Variables summarized by means are analyzed using a Mann-Whitney U test. Reported p-values are two-sided.
